# Understanding the Real Needs and Expectations of French Patients with Amelogenesis Imperfecta Through Facebook Content: A Qualitative Thematic Analysis

**DOI:** 10.3390/healthcare13212740

**Published:** 2025-10-29

**Authors:** Aurelie Mailloux, Jérôme Dinet, Jules Filloux, Yann Lanuel

**Affiliations:** 1 CHU Reims, 51092 Reims, France; aurelie.mailloux@univ-reims.fr; 2 Laboratoire Lorrain de Psychologie et Neurosciences de la Dynamique des Comportements (2LPN), University of Lorraine, 54000 Nancy, France; 3 Laboratoire LCOMS, University of Lorraine, 57000 Metz, France; jules.filloux1@etu.univ-lorraine.fr (J.F.); yann.lanuel@univ-lorraine.fr (Y.L.)

**Keywords:** rare disease, amelogenesis imperfecta (AI), social support, online social network, well-being

## Abstract

**Background and Objectives**: Facebook groups have become support spaces for people with rare diseases such as amelogenesis imperfecta (AI). While their potential for revealing patient needs is recognized, no systematic analysis has been conducted in France. This study aims to better understand the psychological and practical needs of French AI patients by analyzing interactions within a dedicated Facebook group. **Methods**: A semantic and thematic analysis was conducted on 881 texts (39,647 words) from the French Facebook group Amelogenesis Imperfecta. A custom tool, TEXTRA©, and IRaMuTeQ© software were used for analysis, including similarity analysis (lexical co-occurrences), Descending Hierarchical Classification (DHC), Correspondence analysis to reveal discourse structures. **Results**: Correspondence analysis revealed two main discourse trends: individual experiences (symptoms, treatment logistics, and medical engagement) and collective narratives (focused on awareness, mobilization, and institutional recognition). DHC identified four thematic classes: (a) difficulties accessing healthcare, (b) genetic framing and family implications, (c) dental symptoms and treatment experiences, and (d) community advocacy. These findings highlight how the group fosters emotional support, peer exchange, and empowerment. **Conclusions**: Online communities play a vital role in supporting patients with rare diseases. This study shows that the analysis of user-generated content can guide improvements in clinical practice, psychosocial support, and health policy.

## 1. Introduction

The rise in social networks has transformed the Internet into a participatory space where users actively share and exchange experiences, especially in health-related contexts [1,2,3]. Facebook, with over 3 billion monthly active users, is widely used for health support and communication, including those affected by rare diseases [4]. In recent years, health researchers have increasingly analyzed online networks like Facebook to understand care delivery, clinician collaboration, and patient behavior [5,6,7]. For patients, especially those with rare diseases, online communities offer practical knowledge exchange, peer support, and a space to share personal stories, often with a sense of safety and emotional distance [8,9,10]. Despite this growing body of research, little attention has been given to the content exchanged in Facebook groups for patients with amelogenesis imperfecta (AI), particularly in Western Europe.

### 1.1. Amelogenesis Imperfecta (AI) and Its Consequences

**Figure 1:**
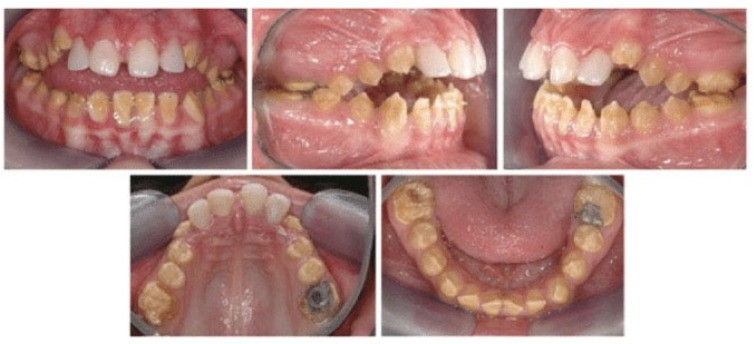
Intraoral photos of an amelogenesis imperfecta patient, issued from [27].

Amelogenesis imperfecta (AI) is a rare genetic disorder affecting tooth enamel development in both primary and permanent teeth (Figure 1) [11]. It results in fragile, discolored, and sensitive teeth, often accompanied by malocclusions, pain, and increased risk of cavities [12]. AI stems from mutations in genes involved in enamel formation, such as AMELX, ENAM, and FAM83H. Beyond physical symptoms, AI significantly impacts psychosocial well-being. Studies show lower quality of life among AI patients due to esthetics, tooth sensitivity, and functional limitations [13,14,15]. Many patients, especially children and adolescents—report social anxiety, bullying, and self-consciousness about their appearance [16,17,18]. Parents, too, may experience guilt or distress related to the hereditary nature of the condition [19,20]. Early, multidisciplinary care, including psychological support, is essential to prevent long-term harm [21].

Several studies show that individuals with AI report significantly lower quality of life than healthy controls, mainly due to esthetic concerns, tooth sensitivity, pain, and functional issues [13,14,15,22,23]. AI is also associated with social anxiety and avoidance, with patients experiencing greater distress related to their oral condition [16]. Younger patients tend to fear negative evaluation more [17], and a UK survey found that 76% of patients aged 5–17 were dissatisfied with their appearance and struggled with social awareness [18]. These effects on self-esteem persist into adulthood [23], and a 15-year follow-up revealed emotional fatigue and strained relationships with healthcare providers when AI is left untreated [24]. Moreover, children and adolescents with esthetic dental malformations like AI are at higher risk of bullying, which negatively impacts academic performance, self-esteem, and social engagement [25,26]. Psychological effects also extend to parents, who often experience guilt and shame related to passing on the hereditary condition, highlighting the need for emotional support for families [14,19,20].

In other words, psychological aspects of quality of life, which is a common feature in patients suffering from many kinds of enamel anomalies, are very important and can affect the life of all patients, from childhood to the elderly. Early, empathetic, and multidisciplinary care (including psychological and social support) greatly reduces long-term harm. Patient testimonials reinforce the need for esthetic and functional treatment, not just for oral health but also for emotional well-being [16,17,21].

### 1.2. Advantages of Online Social Network for Patients

Facebook support groups are particularly valuable for individuals with rare diseases like AI, offering peer connection, shared knowledge, and emotional support across geographic distances [28,29]. The accessibility, semi-anonymity and perceived privacy of the platform encourage open and emotionally rich exchanges that may not surface in clinical settings. Analyzing Facebook content offers several advantages: access to authentic patient perspectives often missing from traditional research [30]; identification of support gaps, misinformation, and common concerns [31]; real-time insights into emerging trends or shifting sentiment; and opportunities for improved patient engagement, satisfaction tracking, and healthcare communication [6,32]; Informing policy and product development in a patient-centered way [33,34]; Cost-effective data collection at scale [35]. In this context, our study aims to analyze the discursive structure of a French Facebook group dedicated to patients and families affected by AI, highlighting how digital platforms shape collective and individual experiences of living with a rare dental disorder.

### 1.3. Limitations of Previous Studies and Our Objectives

Although all these previous studies have yielded valuable insights, several limitations remain, highlighting the need for further research specifically focused on amelogenesis imperfecta: To date, no study has been conducted with French patients and their families, which represents a significant gap in the literature and limits our understanding of how amelogenesis imperfecta is experienced and managed within the French healthcare and cultural context. The management of amelogenesis imperfecta (AI) varies significantly across cultural and healthcare contexts [14,27,36,37]. In high-income countries, treatment often involves early diagnosis, multidisciplinary care (including pediatric dentists, orthodontists, prosthodontists, and geneticists), and access to advanced restorative materials and techniques such as full crowns, veneers, or implants. In contrast, in lower-resource settings, AI treatment can be limited by lack of access to specialized care, financial constraints, and low public awareness of the condition. Moreover, cultural perceptions of dental esthetics and pain tolerance can influence how patients seek care and adhere to treatment plans. Finally, stigma and psychological distress related to visible dental anomalies may be addressed differently across societies, with some cultures showing greater sensitivity to facial appearance than others. These disparities highlight the importance of context-sensitive, equitable treatment strategies and the need for global collaboration to ensure that patients with AI receive timely, adequate care regardless of their cultural or economic background.

Moreover, although several authors acknowledge that Facebook content can offer valuable insights into the actual needs and expectations of patients, no rigorous study has yet been conducted to systematically analyze this material [38]. While data collection in this field is often unidimensional (focusing primarily on economic, educational, or occupational variables), our present study is grounded in a multidimensional approach that integrates semantic, thematic, and contextual analyses to more fully capture the complexity of patient experiences.

In other words, the main objective of our study is to gain a deeper understanding of the actual needs and psychological factors that affect French patients living with amelogenesis imperfecta (AI), through a rigorous analysis of all the content exchanged within a dedicated Facebook group for patients and their members’ family.

## 2. Materials and Methods

To explore the actual needs and mental representations of French patients and their families regarding amelogenesis imperfecta, we conducted a semantic and thematic analysis of a corpus derived from a dedicated Facebook group. Lexical proximity between key terms was examined using similarity analysis, while thematic patterns were identified through a Descending Hierarchical Classification (DHC) based on the Reinert method [39,40,41,42]. The analysis was performed on a corpus comprising 881 texts, totaling 39,647 words and 3276 unique word forms. All stages of the research process were conducted by the authors without the use of artificial intelligence tools. Data collection, corpus preparation, and correspondence analysis were performed by AM, JF, JD, YL. Interpretation of the lexical classes and thematic analysis were conducted collaboratively by JD, AM, and manuscript drafting and revision were carried out by AM, JD, YL, JF.

### 2.1. The French Facebook Group Amelogenesis Imperfecta

The French Facebook group Amelogenesis imperfecta (https://www.facebook.com/groups/258243454375660/ (accessed on 25 September 2025)) was created in August 2016, are 535 members and the association was created two years later (in March 2018). This French Facebook group is private and is available as a safe space and resource for patients and their families to have the conversations that matter to them. This Facebook group consists of users based in France, French-speaking and the minimum age of the participants is 18. There is no official healthcare professional associated with this discussion group; however, very few practicing dentists answer some questions. The text in the homepage is the following: “*Information and exchange on amelogenesis imperfecta—for patients and their families. In this group, people closely affected by the disease will be able to share information, testify, support, and advise each other. We hope they will also be able to join forces to raise awareness of the disease, better defend their rights and raise public awareness*”.

### 2.2. Design and Tools

**Figure 2:**
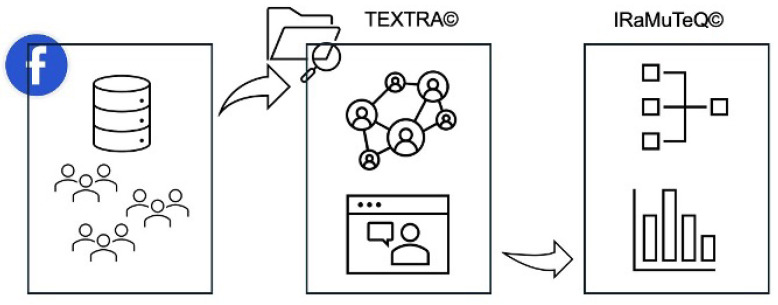
Workflow of data analysis: From Facebook group collection to automated extraction TEXTRA© and thematic analysis IRaMuTeQ©.

Thematic analysis is one of the most prominent methods used for the analysis of message content [28,29,30,31]. Thematic analysis assumes that the recorded messages themselves (i.e., the texts) are the data, and codes are developed by the investigator during close examination of the texts as salient themes emerge inductively from the texts. For our study, two specific software have been used (Figure 2).

#### 2.2.1. Data Extraction

The corpus was extracted using a custom-designed software called TEXTRA©, specifically created for the purposes of this study. Developed by a research team at the University of Lorraine, TEXTRA© was designed to enable the automatic extraction of all data from a Facebook group. This includes the retrieval of posts, comments, replies to comments, and user-related metadata. By capturing the full structure of interactions within the group, encompassing both content and engagement dynamics, TEXTRA© ensured a comprehensive and faithful representation of the discursive material shared by members. Prior to the development of this innovative tool, corpus extraction had to be performed manually, which was both time-consuming and prone to errors. TEXTRA© thus represents a significant methodological improvement, enhancing both efficiency and data accuracy.

#### 2.2.2. Data Analysis

The second software used is IRaMuTeQ©: This software, developed by a research team in Toulouse University, allows for detailed statistical analysis of the text corpus, including classic text analysis, specificity analysis, similarity analysis, and word cloud. IRaMuTeQ© is a GNU GPL(v2) licensed software that provides users with statistical analysis on text corpus and tables composed by individuals/words It is based on R software (version 5.3.1) and on the Python language (version 3.14.1). This software provides the users with different text analyses, either simple ones, such as the basic lexicography related to lemmatization and word frequency; or more complex ones such as Descending Hierarchical Classification, post hoc correspondence factor analysis and similarity analysis. In our study, two text analyses provided by IRaMuTeQ© have been used:

- Similarity analysis. This analysis, based on graph theory, is often used by social representations researchers. It allows to identify the words co-occurrences, providing information on the words connectivity thus helping to identify the structure of a text corpus content. It also allows us to identify the shared parts and specificity according to the descriptive variables identified in the analysis.
- Method of Descending Hierarchical Analysis (DHA). The content and text segments are clustered according to their vocabularies and distributed according to the reduced forms frequencies. Using matrices that cross reduced forms with textual segments, the DHA method allows you to obtain a definitive classification. It is aimed at obtaining clusters with similar vocabulary within, but different from other segments. A dendrogram will be displayed showing clusters relations. The software calculates descriptive results of each cluster conforming to its main vocabulary (lexic) and words with asterisk (variables). Furthermore, it provides the users with another way of presenting data, derived from a correspondence factor analysis. Based on the chosen clusters, the software calculates and provides the most typical TS of each cluster, giving context to them.

### 2.3. Ethics

The study was conducted in accordance with the Declaration of Helsinki and was approved by the Ethics Committee of Reims hospital (Study Protocol No. RN25016).

## 3. Results

To better understand the effective needs, opinions and representation of patients and families interested in amelogenesis imperfecta, an analysis of the semantic structure of the corpus issued from the Facebook group related to the French association and a thematic analysis based on a Descending Hierarchical Classification using the Reinert method analysis were performed. The analysis was based on a corpus of 881 texts, totaling 39,647 words and 3276 unique forms.

### 3.1. Similarity Analysis and Clustering

**Figure 3:**
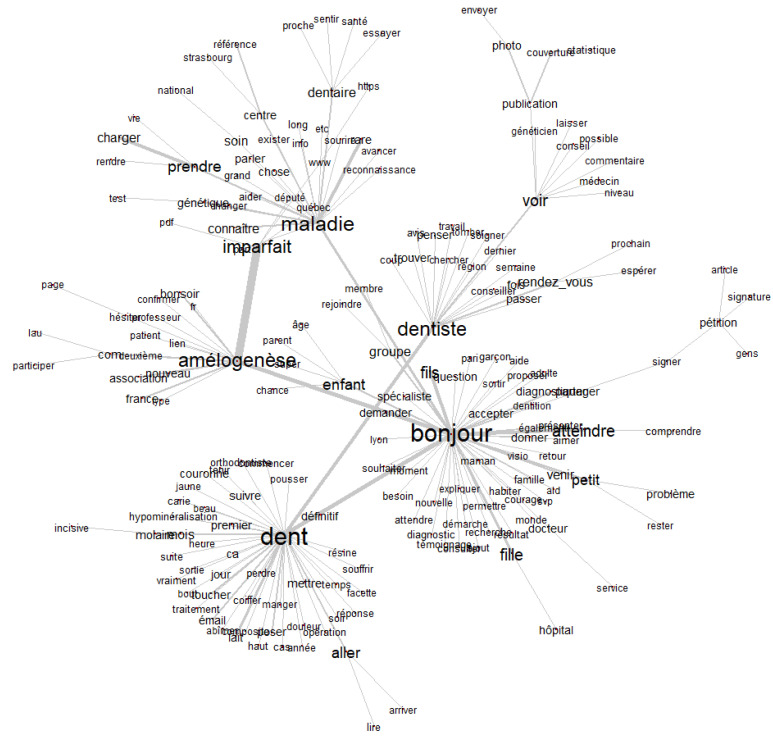
Similarity analysis of content clusters from the Facebook group using IRaMuTeQ©.

The similarity analysis conducted using IRaMuTeQ© to visualize significant co-occurrences between lexical forms in the corpus extracted from the French Facebook group amelogenesis imperfecta show several interesting results. The resulting graph (Figure 3) reveals three well-defined lexical clusters that reflect how participants discuss and experience amelogenesis imperfecta (AI):

- The word “dent” (tooth) forms the central lexical node, with numerous connections to everyday terms such as “fils” (son), “fille” (daughter), “mettre” (put), “perdre” (lose), “petit” (small), “problème” (problem), indicating real-life, family-centered experiences related to dental symptoms. This cluster is strongly linked to “dentiste” (dentist), which is in turn connected to terms like “traitement” (treatment), “rendez-vous” (appointment), “avis” (opinion), “diagnostiquer” (diagnose), highlighting the central role of healthcare and professional consultations.
- A second semantic hub is organized around “maladie” (disease), connected to “génétique” (genetic), “enfant” (child), “rare”, “connaître” (to know). This reflects a medicalized view of the condition, often framed in terms of inheritance, diagnosis, and rarity. The presence of terms such as “pétition” (petition), “centre” (center), “changer” (change), “donner” (give) suggests a broader collective or advocacy dimension, possibly parental or patient-led efforts seeking recognition or policy change.
- Finally, the word “amélogenèse” (amelogenesis) appears in direct connection with “imparfait” (imperfect), “atteindre” (affect), “association”, reflecting the shared awareness of the condition’s name and the need for information-sharing and support, as seen in related terms like “photo”, “groupe”, “partager” (share).

Overall, the graph related to the correspondence analysis of clusters highlights a dual discourse structure: one rooted in personal and familial experiences, the other centered on healthcare, diagnosis, and advocacy, typical of narratives surrounding rare and hereditary disorders.

### 3.2. Correspondence Analysis and Factorization

**Figure 4:**
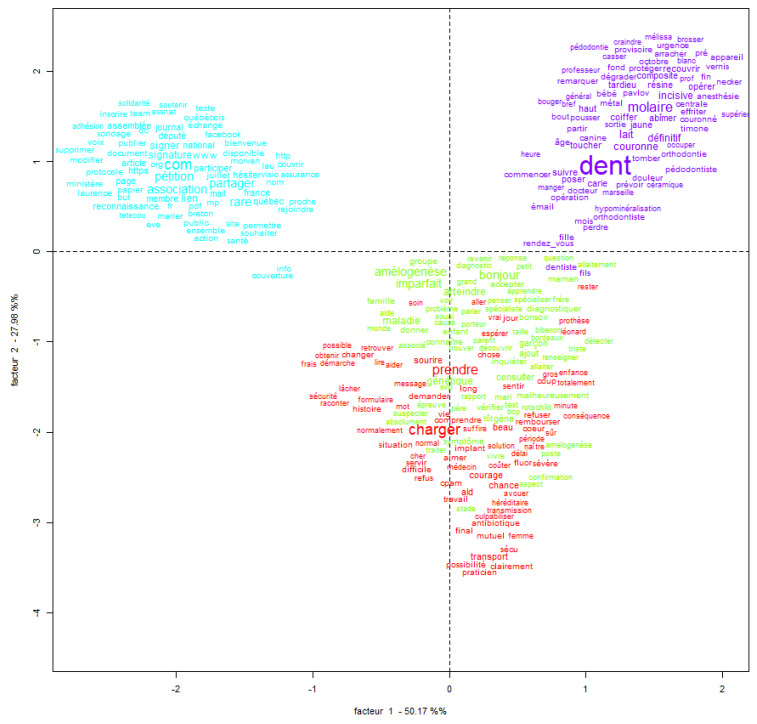
Correspondence mapping of clusters derived from Facebook group content via IRaMuTeQ©.

The correspondence analysis of clusters has two main axes (Figure 4):

- Factor 1 (horizontal axis): Explains 49.34% of the variance. This factor 1 distinguishes Clinical vs. Activist Dimension. The right side (Red/Green/Blue) focuses on clinical, genetic, and symptom-based discourse; the left side (Purple) focuses on community, activism, media, and public communication This axis opposes individual/medical experience to collective/social mobilization.
- Factor 2 (vertical axis): Explains 23.26% of the variance. This factor 2 distinguishes Technical vs. Personal/Descriptive Dimension. The top (blue) is related to lexicon rich in concrete dental symptoms and care (e.g., “molaire”, “résine”, “douleur”); the bottom (red/green) emphasizes institutional, procedural, or psychological aspects (e.g., “rendez-vous”, “médecin”, “traitement”, “hôpital”).

Together, these two axes explain 72.6% of the variance which is a solid basis for interpretation. The correspondence analysis highlights two primary axes organizing the corpus’ discourse. The first axis (49.34%) distinguishes a medical and individual discourse—ranging from personal symptoms (Class 3) to institutional navigation (Class 1), from a collective, activist discourse centered on public visibility and community mobilization (Class 4). The second axis (23.26%) separates a technically descriptive discourse focused on dental symptoms from more abstract, clinical, or genetic considerations (Classes 1 and 2). Notably, the activist class (Class 4) is the most distinct, forming its own quadrant and suggesting that community-driven narratives are lexically and semantically distant from medically oriented ones. Class 2 occupies a central position, bridging scientific understanding with patient-family experience.

### 3.3. Thematic Analysis of Comments and Exchanges

**Figure 5:**
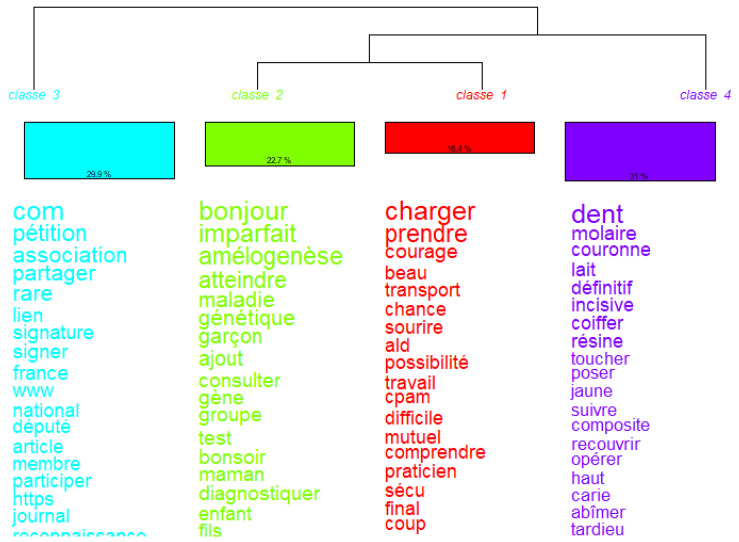
Dendrogram of clusters from Descending Hierarchical Analysis (DHA) using IRaMuTeQ©. Branch divisions represent the structure of lexical cluster differentiation.

The Descending Hierarchical Classification dendrogram from IRaMuTeQ© (Figure 5), based on the Reinert method analysis, where the corpus is divided into thematically consistent lexical classes based on word co-occurrence, shows four disinct classes:

- Class 1 (in red): Medical Professionals and Access to Care (21%), with the following top words: “spécialiste”, “docteur”, “rendez-vous”, “dentiste”, “médecin”, “hôpital”, “centre”, “travail”, “connaître”, “répondre”. This class reflects a discourse focused on accessing the healthcare system: it centers around interactions with specialists, doctors, and institutions. The presence of location-specific terms (e.g., Lyon, Bordeaux, Montpellier) and verbs like répondre, prendre suggest issues related to appointments, delays, and logistics. We propose to label this class “Healthcare navigation and access difficulties”.
- Class 2 (in green): Genetic and Clinical Nature of the Disease (19%), with the following top words: “génétique”, “maladie”, “imparfaite”, “gène”, “enfant”, “atteindre”, “test”, “amélogenèse”, “transmettre”. This class reflects a biomedical perspective of the condition. It includes genetic terms and refers to the transmission, testing, and emotional impact of amelogenesis imperfecta. Words like “chance”, “vivre”, “courage” suggest some existential or emotional framing of the diagnosis. We propose to label this class “Genetic disease framing and family impact”.
- Class 3 (in blue): Description of Dental Symptoms (29.1%), with the following top words: “dent”, “molaire”, “couronne”, “lait”, “toucher”, “poser”, “incisive”, “fils”, “carie”, “abîmer”, “jaune”. This is the most concrete and symptom-centered class, focusing on dental manifestations and terminology. It includes references to deciduous teeth (“lait”), materials (“résine”), stages (“sortie”), and treatments (“couronne”). This suggests parents or individuals are detailing clinical signs and treatment attempts. we propose to label this class “Dental symptoms and treatment experiences”;
- Class 4 (in purple): Advocacy, Community, and Visibility (31%), with the following top words: “com”, “pétition”, “partager”, “association”, “signature”, “signer”, “reconnaissance”, “député”, “participer”, “article”. This class stands apart as a collective, activist discourse. It includes words related to online engagement, petitioning, awareness-raising, and media participation. The use of “com”, “admin”, “signature”, “député” suggests efforts to gain recognition for the disease and mobilize institutional support. We propose to label this class “Community mobilization and advocacy”.

Classes 3 and 4 are the most distinct, i.e., they split early in the tree. Classes 1 and 2 are closer lexically, suggesting that navigating the medical system and understanding the genetic basis of the disease often appear together in the same segments of discourse.

## 4. Discussion

This study highlights how analyzing Facebook group interactions offers valuable insights into the everyday experiences and collective concerns of people with amelogenesis imperfecta (AI). By combining automated and manual analysis methods, we captured the complex realities of living with AI, including challenges in care access and the importance of community advocacy. These findings underscore the potential of online patient groups to inform healthcare and guide future research.

### 4.1. Methodological Considerations

Analyzing posts and exchanges in a patient Facebook group captures spontaneous, lived experience, and emergent concerns that structured interviews or questionnaires may miss. Therefore, online group data provide ecological validity to understand everyday needs, emotions, and coping strategies among people with rare conditions such as amelogenesis imperfecta (AISocial media archives allow access to a large volume of messages and interactions over time, allowing detection of persistent themes (for example repeated access problems, advocacy efforts) and short-term events (treatment crises, appeals). Automated lexical methods (correspondence and similarity analyzes, descent hierarchy classification) should be combined with manual reading or thematic triangulation to improve validity compared to using purely manual coding or automated approaches alone [43]. Facebook group participants are a self-selected subset of the AI population (maybe more digitally engaged, with homogeneous age, etc.). The findings therefore reflect the concerns of a minority of active group members and their families. This does not include the entire AI population or those who avoid online sharing [43,44,45,46]. Future research would benefit from cross-platform comparisons (Instagram, dedicated forums) to provide a more complete understanding of patient experiences. Even if group content is publicly viewable, ethical guidelines recommend careful consideration of consent, anonymization, and potential harm to participants if quoted verbatim [47]. Archival social media research raises questions about whether users expect their posts to be used in research.

The use of TEXTRA, a custom tool for automated Facebook data extraction, ensured a comprehensive and unbiased corpus collection, addressing the limitations of manual selection in previous studies [3]. This rigor, combined with the analytical capabilities of IRaMuTeQ©, allowed us to capture authentic unfiltered patient narratives, including emotions like fear, frustration, and vulnerability—commonly reported in rare diseases [46,47,48].

### 4.2. Discussion of Results and Perspectives

A cumulative explained variance of at least 60% is considered acceptable for a valid interpretation of the axes in factor analysis [49]. In our study, the first two axes account for 72.6% of the total variance, which is more than sufficient for a structural interpretation of the corpus. The correspondence analysis performed on the content extracted from the Facebook group for French patients and families concerned with amalogenesis imperfecta (AI) provides a meaningful structural interpretation of the corpus, with the two primary axes accounting for 72.6% of the total variance, a robust analytical foundation. The first axis (49.34%) reveals a fundamental contrast between discourses rooted in medical and individual experience (notably Classes 1 and 3) and those centered on collective action and public advocacy (Class 4). This suggests a divergence between personal narratives focusing on symptoms, care logistics, and medical participation, and a broader discourse aimed at visibility, mobilization, and institutional recognition. The second axis (23.26%) differentiates a technically descriptive lexicon, particularly related to dental manifestations (Class 3), from a more abstract, clinical and genetic discourse (Classes 1 and 2). This gradient underscores the multifaceted nature of the corpus, ranging from concrete symptomatology to conceptual and etiological framing of the condition.

The Descending Hierarchical Classification (DHC) of the corpus revealed four distinct lexical classes: (1) healthcare navigation and access difficulties (21%); (2) genetic framing and family impact (19%); (3) dental symptoms and treatment experiences (29,1%); and (4) community mobilization and advocacy (31%), the latter being the most prominent class for patients and families. The correspondence analysis showed a divergence between personal narratives centered on daily symptoms, care logistics, and clinician encounters, and a broader discourse oriented to visibility, mobilization, and institutional recognition. The study confirms that Facebook support groups provide social, informational, and emotional support: peer friendship, practical knowledge exchange, coping strategies, and empowerment.

The difficulties in accessing specialized dental care, financing restorative treatments, and navigating referral pathways are common themes in the literature on rare diseases [1,50,51]. For AI specifically, the high cost and limited availability of cosmetic and restorative dental options are recurrent complaints in clinical and patient reports. Therefore, online groups function as a practical knowledge repository for care pathways and clinician recommendations. The importance of genetic discourse and family narratives aligns with studies showing that rare hereditary conditions impact family identity, reproductive decision making, and parental guilt [2,46].

The focus on sensitivity, enamel fragility, esthetic concerns and treatment sequencing (childhood vs. adult rehabilitations) is consistent with AI clinical and quality of life research demonstrating substantial functional and psychosocial sequelae from enamel defects. The study’s finding that advocacy/visibility is the main theme reflects broader evidence that online communities for rare diseases commonly evolve from peer support to collective action: awareness campaigns, fundraising, and lobbying for recognition of the health system [44,45]. Together, these findings emphasize the complexity of patient experiences that span medical, emotional, and social domains. Healthcare providers and policymakers should consider this multilayered reality when designing services, ensuring they address not only clinical needs, but also access challenges and psychosocial support. Some future research directions can be planned based on our findings. For instance, because our study captured a snapshot in time and because online communities are dynamic and often shift focus as membership grows or as collective goals develop, it could be interesting to conduct a longitudinal study of discourse evolution within the same Facebook group to observe changes over time (e.g., from support to advocacy). In the same way, because different platforms afford different communication styles and audience reach, which could influence the framing of issues (e.g., more activism on Twitter vs. emotional support on Facebook), it could be relevant to compare discourse structures across different platforms (e.g., Facebook vs. Reddit vs. Twitter/X). And finally, because health system structures influence care access, contrasting discourse may reflect systemic differences in coverage, diagnosis, and support, it could be very interesting to compare patient discourse in AI Facebook groups across countries (e.g., France vs. Canada vs. the US).

Although our study provides novel insights into the experiences of French patients with amelogenesis imperfecta (AI) through social media discourse, several limitations must be acknowledged. First, the analysis was based on a single Facebook group, which may not fully represent all individuals affected by AI in France or those who do not use social media. Second, the data reflect self-selected participants and spontaneous posts, which may emphasize more vocal or motivated members and underrepresent those with different perspectives or limited digital literacy. Third, linguistic nuances and context may be partially lost when interpreting textual data outside their conversational setting. Consequently, while the findings reveal key themes relevant to patients’ lived experiences, their generalizability is limited to online, self-supporting communities and may not extend to the broader population of AI patients. Nonetheless, the thematic patterns identified, healthcare access challenges, emotional coping, and collective advocacy, are consistent with evidence from other rare-disease contexts, suggesting that similar psychosocial dynamics may be observed in comparable groups internationally.

## 5. Conclusions

This study demonstrates that Facebook groups provide unique access to the concrete needs of patients with rare diseases such as amelogenesis imperfecta (AI), revealing clinical, psychological, and social challenges often underrepresented in traditional research. The exchanges analyzed highlight the difficulties in accessing care, the critical need for emotional support and information, and collective mobilization to increase recognition of the disease. These findings underscore the importance of using patient-generated data to tailor medical care, public health policies, and psychosocial support systems. A follow-up qualitative study (surveys and interviews) is planned to refine our understanding of lived experiences and assess the real-world impact of these online communities on the quality of life of patients and their families.

## Data Availability

The data are archived and stored at CHU Reims and can be obtained upon request.
